# Integrated Small Animal PET/CT/RT with Onboard PET/CT Image Guidance for Preclinical Radiation Oncology Research

**DOI:** 10.3390/tomography9020046

**Published:** 2023-03-04

**Authors:** Xinyi Cheng, Dongxu Yang, Debabrata Saha, Xiankai Sun, Yiping Shao

**Affiliations:** 1 Department of Radiation Oncology, University of Texas Southwestern Medical Center, Dallas, TX 75390, USA; 2 Simmons Comprehensive Cancer Center, University of Texas Southwestern Medical Center, Dallas, TX 75390, USA; 3 Department of Radiology, University of Texas Southwestern Medical Center, Dallas, TX 75390, USA; 4 Advanced Imaging Research Center, University of Texas Southwestern Medical Center, Dallas, TX 75390, USA

**Keywords:** onboard PET/CT image, PET/CT/RT, preclinical radiotherapy research, small animal PET

## Abstract

We have integrated a compact and lightweight PET with an existing CT image-guided small animal irradiator to enable practical onboard PET/CT image-guided preclinical radiation therapy (RT) research. The PET with a stationary and full-ring detectors has ~1.1 mm uniform spatial resolution over its imaging field-of-view of 8.0 cm diameter and 3.5 cm axial length and was mechanically installed inside the irradiator in a tandem configuration with CT and radiation unit. A common animal bed was used for acquiring sequential dual functional and anatomical images with independent PET and CT control and acquisition systems. The reconstructed dual images were co-registered based on standard multi-modality image calibration and registration processes. Phantom studies were conducted to evaluate the integrated system and dual imaging performance. The measured mean PET/CT image registration error was ~0.3 mm. With one-bed and three-bed acquisitions, initial tumor focused and whole-body [^18^F]FDG animal images were acquired to test the capability of onboard PET/CT image guidance for preclinical RT research. Overall, the results have shown that integrated PET/CT/RT can provide advantageous and practical onboard PET/CT image to significantly enhance the accuracy of tumor delineation and radiation targeting that should enhance the existing and enable new and potentially breakthrough preclinical RT research and applications.

## 1. Introduction

Combined PET/CT functional and anatomic images are ubiquitously used in clinical radiotherapy (RT) for tumor diagnosis and delineation to guide the treatment plan and access the therapeutic effects. However, in the field of preclinical radiation research, all existing image-guided small animal irradiator systems are only equipped with onboard CT without PET due to various technical reasons. Although optical or other modality imaging technology can provide some functional information of the tumor, for most radiation oncology studies, particularly for those with orthotopically implemented animal tumor models, PET is still the modality that can provide the desired quantitative, functional/biological/molecular image to substantially improve the accuracy of radiation targeting to reduce the radiation margin for increasing the dose to the tumor and sparing the surrounding normal tissues. The lack of preclinical PET/CT image-guided RT capability has severely limited the precision of animal RT study to accurately investigate radiation’s biological effects and, more importantly in the era of translational RT research, the value to translate the findings from preclinical research to clinical applications or vice versa.

We recently developed a compact and lightweight small animal positron emission tomography (PET) with uniform, high spatial resolution across its imaging field-of-view (FOV) that is suited for integration with a cone-beam computed tomography (CBCT, or CT for short) image-guided small animal radiation therapy (RT) irradiator (CT/RT) [1,2,3]. Although functional and anatomical images can be acquired by the standalone PET and the CT inside the irradiator and the dual-modality images can be co-registered through software [4,5,6], such offboard PET/CT imaging requires the transportation and reposition of the animal over separated modalities that will lengthen the scan time and be prone to dual-modality alignment and associated image registration errors [7,8]. On the other hand, the integrated onboard PET/CT/RT can streamline the process of animal positioning, dual-image acquisition, and data processing to minimize the scan time and alignment error, simplify the workflow, and even permit motion compensated or other advanced image-guided RT applications with the onboard PET/CT imaging [9,10].

In this study, we report the integration of the PET with an existing CT/RT and the performance evaluation of this integrated preclinical PET/CT/RT for onboard PET/CT image-guided preclinical RT research.

## 2. Materials and Methods

### 2.1. PET

**Figure 1:**
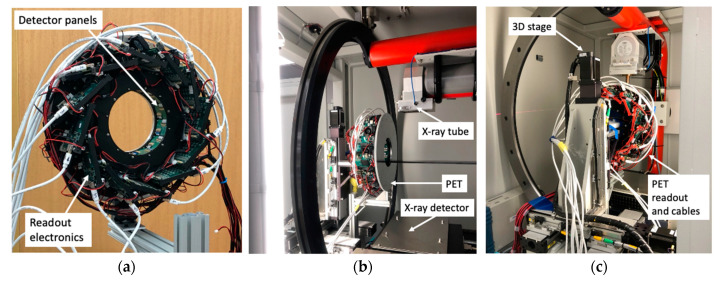
Photos of (**a**) the assembled PET gantry attached with LVDS and power supply cables and (**b**) and (**c**) the front and back views of the installed PET gantry inside the small animal irradiator.

As shown in Figure 1a, the compact and lightweight PET is suited to be installed inside an existing small animal CT/RT for mechanical integration. The following are summaries of its specification and performance [1].

The PET consists of a ring of 12 detector panels in a dodecagon configuration. Each detector panel has a 30 × 30 array of 1 × 1 × 20 mm^3^ Ce-doped Lutetium-Yttrium Oxyorthosilicate (Lu_0.6_Y_1.4_SiO_0.5_:Ce, LYSO) scintillators. Each end of the scintillator array is optically coupled to an 8 × 8 silicon photomultiplier array (model MPPC S13361-2050-08, Hamamatsu Photonics K.K., Shizuoka, Japan) for depth-of-interaction (DOI) measurement based on the dual-ended readout [11]. All six scintillator surfaces were lapped with 0.03 mm grade to provide balanced light output and good DOI resolution. Optical reflective films (ESR, 3M Corp, Saint Paul, MN, USA) with 0.06 mm thickness were used between scintillators to prevent inter-scintillator optical crosstalk and enhance the light output of each crystal. Each MPPC active pixel size is 2 × 2 mm^2^, with nominal operational bias and dark count rate around 54.4 v and ~500 Kcps.

The outer diameter and axial length of the PET gantry are 33.0 cm and 11.0 cm, respectively, with a 11.0 cm diameter animal port. The total weight of the gantry is 6.5 kg, which includes the PET detector ring, front-end readout electronics boards, air-low fans, 3D-printed packaging holders, and metal plate for installation. The imaging field-of-view (FOV) is 8.0 cm in diameter and 3.5 cm axial extent, with an 11.0 cm diameter animal port. The mean spatial resolutions along radial, tangential, and axial directions were measured as 1.30, 1.18, and 0.96 mm with ~11.8% uniform activity background. A ~1.1 mm uniform spatial resolution was achieved within 20 mm FOV radius without the resolution recovery, while the same ~1.1 mm uniform spatial resolution within 30 mm FOV radius can be achieved by including the resolution recovery with a ~1.0 mm full-width-half-maximum (FWHM) Gaussian [12]. The maximum sensitivity at the center of FOV was ~1.8% with a 350–650 keV energy window.

### 2.2. Integration of PET and CT/RT

With the mechanical support of off-the-shelf metal bars, the PET gantry was stationarily installed inside the CT/RT (X-RAD 225Cx, Precision X-ray Irradiation, Madison, CT, USA) in a tandem PET/CT imaging configuration (Figure 1). Laser beams were used to ensure that the orientations and centers of both the PET and CT imaging FOVs were closely aligned with each other. To minimize the impact of X-ray radiations, a 10 mm thick aluminum alloy metal plate was placed at the end of the PET gantry that faced the incoming scattered X-rays. For data transmission, signal synchronization, and system command, twelve low-voltage-differential-signals (LVDS) cables were used to connect 12 front-end detector readout boards to the system electronics board sitting outside the CT/RT. The PET data acquisition computer was also placed outside and next to the CT/RT control console. By placing the system electronics board and acquisition computer outside the CT/RT, we eased the problem of the limited interior CT/RT space and minimized the heat generated from PET components that could raise the temperature inside the CT/RT and affect the detector performance.

**Figure 2:**
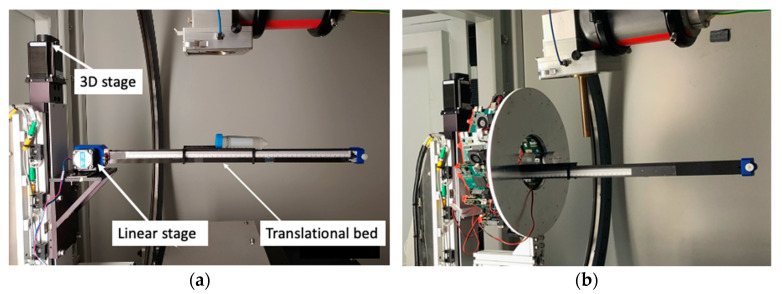
Photos of (**a**) the translational bed driven by a linear stage and (**b**) the integrated PET/CT for dual modality imaging.

To enable the translation of an animal between PET and CT for dual imaging acquisitions, an additional linear translational bed was constructed and attached to the existing 3D stage to extend the horizontal translation range that was limited by the original 3D bed motion mechanical system. This additional bed consisted of a thin carbon-fiber curved plate on plastic lightweight rails driven by a step motor for linear translational motion (Figure 2). The size and thickness of the plate were 15.5 cm long, 5.0 cm wide, and 2 mm thick, which had approximately the same attenuation effect to CT image as that with the original animal bed.

PET data were independently acquired and processed with the system’s PC computer. A user interface based on MATLAB (MathWorks, Natick, MA, USA) was developed to control the motion of the linear translational animal bed, start and stop the data acquisition, and monitor the detector performance. Acquired data were processed offline for data calibration, selection, and correction.

### 2.3. PET/CT Coordinate System Alignment for Dual-Modality Image Registration

An existing method was applied to align the PET and CT coordinate systems for image registration [13]. It was based on rigid-body transformation of dual-modality fiducial markers to measure the misalignment between the PET and CT coordinate systems and transform the original PET coordinate system to accurately align it with the CT coordinate system.

In this study, five independent PET and CT scans were conducted to measure the dual modality positions of a ^22^Na point source with 251 kBq activity [MMS03 Multimodal Imaging Source, Eckert & Ziegler Isotope Products, Valencia, CA, USA]. The physical ^22^Na radioactivity material was sealed at the center of a plastic cube with 10 × 10 × 10 mm^3^ volume. The spherical radioactivity source with around 0.25 mm diameter was precisely at the geometric center of the cube and was used as the CT-measured point source position. The PET-measured point source position was calculated from the intensity-weighted centroid of the corresponding reconstructed PET image. For each PET/CT scan, the point source was placed at a different non-coplanar position inside the PET and CT image FOVs, which was achieved by inserting the point source into a size-matching cubic hole at the surface of a holder that was 3D-printed with low density material and a hollowed center to minimize the attenuation. The holder had five such cubic holes at its different surfaces and locations for five different PET/CT scans. The holder was rigidly fixed on the bed during all five PET/CT scans. The differences among corresponding PET-measured and CT-measured point source positions were used to measure the misalignment between the PET and CT coordinates and, correspondingly, to transform and align the PET coordinate system with the CT coordinate system that had its center of FOV (CFOV) at the RT isocenter.

### 2.4. Initial Onboard PET/CT Imaging Study

#### 2.4.1. Phantom Study

An ultra-micro hot-rods phantom (Data Spectrum Corporation, Durham, NC, USA) filled with [^18^F]NaF was used to evaluate the dual-modality acquisitions and the accuracy of the registered PET/CT images. The diameter and length of the phantom insert were 26.0 and 10.0 mm. The diameters of the through holes (rods), which were arranged in six sections within the insert, are 0.75, 1.0, 1.35, 1.7, 2.0, and 2.4 mm, respectively. The phantom was placed at the CFOV of each imaging modality for its acquisition. The PET image was acquired for 30 min with a start radioactivity at 3.8 MBq, 350–650 keV energy window, and 10 ns coincidence timing window. An open-source code (CASToR) based on an ordered subset expectation maximization (OSEM) algorithm was used to reconstruct the image with 10 subsets and 10 iterations [14]. CT image data were acquired from 301 projections of 40 kVp and 5 mA X-rays and were reconstructed with standard a FDK algorithm [15]. PET and CT phantom images were registered with the aligned PET/CT coordinate systems. The accuracy of the image registration was assessed by the difference between the rod centers measured from the PET and CT images.

#### 2.4.2. Animal Study

**Figure 3:**
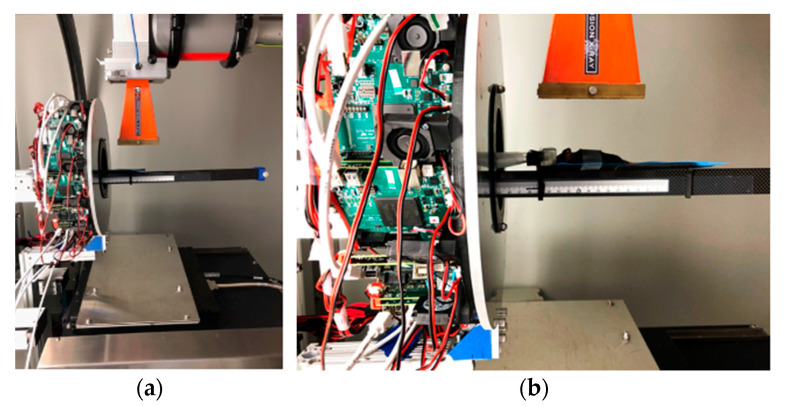
Animal PET/CT imaging study with a mouse at the bed position with its tumor within (**a**) PET FOV and (**b**) CT FOV.

An initial onboard PET/CT animal tumor imaging study was conducted. Figure 3 shows a ~21 g mouse bearing a tumor being placed at the positions for PET and CT imaging acquisitions. The tumor (human lung carcinoma cell, H460), which had been subcutaneously implanted in the hind leg of a female athymic nude mouse, was measured with a size of ~3.9 mm width and ~7.1 mm length at the time of imaging. For PET imaging, ~5.6 MBq of 2-deoxy-2-[^18^F]fluoro-D-glucose ([^18^F]FDG) was injected via the tail vein for data acquisition (25 min) after radiotracer uptake (~30 min). Coincidence events were selected with a 350–650 KeV energy window and a 10 ns time window. The images were reconstructed using CASToR with 4 subsets and 4 iterations, 128 × 128 × 128 matrix, and 0.5 × 0.5 × 0.5 mm^3^ voxel size. For CT imaging, 301 projection data were acquired with 40 kVp and 5 mA X-rays and were reconstructed with standard a FDK algorithm with 350 × 350 × 350 matrix and 0.4 × 0.4 × 0.4 mm^3^ voxel size.

In addition to the one-bed tumor focused image, a whole-body animal PET image was also achieved by acquiring data with the animal being at three sequential bed positions and joining their corresponding reconstructed images based on the known imaging positions. The same data acquisition parameters, data process, and image reconstruction used in the one-bed tumor imaging were applied, except a radioisotope decay correction was also applied to the dataset acquired at each bed position to ensure the similar count statistics among the datasets [16].

## 3. Results

### 3.1. PET and CT Coordinate System Alignment

**Figure 4:**
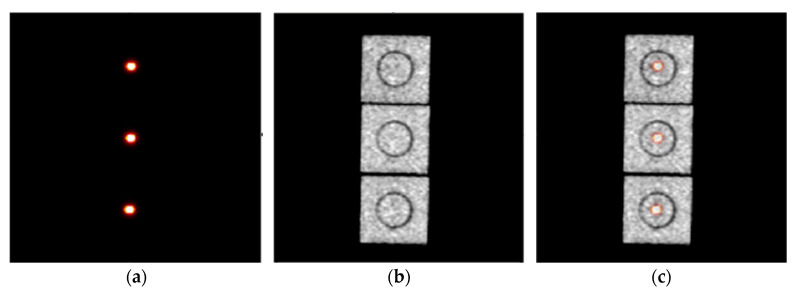
(**a**) PET and (**b**) CT images of the ^22^Na point source at three different FOV positions but viewed through the PET and CT sagittal slices, and (**c**) the corresponding registered PET and CT images of the point source before the PET coordinate system transformation. The difference between the PET and CT measured point source positions was used for PET and CT coordinate system alignment.

Figure 4 shows the measured PET, CT, and PET/CT images of the ^22^Na point source at three different FOV positions. The differences among all five source positions measured between PET and CT were used to calculate the PET/CT coordinate misalignment for PET coordinate transformation and alignment. The registered images show that PET and CT coordinates can be accurately aligned with the described method and procedure.

### 3.2. Phantom Study with Onboard PET and CT Acquisitions

**Figure 5:**
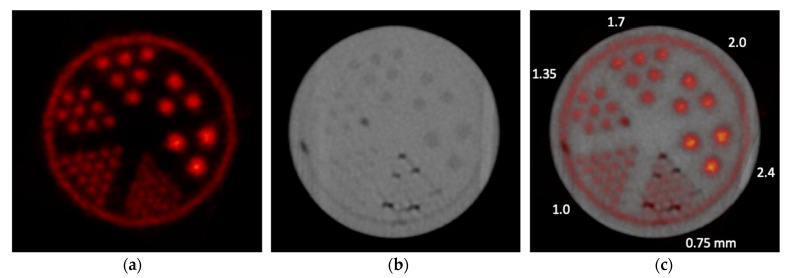
Individually acquired (**a**) [^18^F]NaF PET and (**b**) CT images of the phantom and (**c**) the combined PET/CT image.

Figure 5 shows the [^18^F]NaF PET, CT, and registered PET/CT images of the ultra-micro hot-rods phantom acquired with the onboard PET and CT. For the PET image that is shown in the transformed PET coordinate system, all hot-rods from 1.0 mm to 2.4 mm diameter are clearly separated. It also shows that uniform spatial resolution can be achieved by the PET detectors with the DOI measurement capability that is desired for a compact PET geometry [17]. For the CT image, the image contrast and quality are relatively low due to the use of cone-beam CT. There are also image artifacts at the edge of the phantom due to the additional attenuation from the rails that mechanically support the translational bed (Figure 3), and darker spots in smaller rods and the area between the insert and outer holder of the phantom due to air bubbles produced during the radiotracer filling. Although the contrast and image quality are relatively low, all rods from 1.35 mm to 2.4 mm diameter rods can still be clearly identified and separated. The registered PET/CT image shows that both images are well aligned with each other. The centers of the identified rods were measured with the PET and CT images, and the mean difference among the PET-measured and CT-measured centers was 0.28 ± 0.27 mm, with 0.07 mm minimum and 0.67 mm maximum, which demonstrated that a sufficiently accurate onboard PET/CT image registration can be achieved.

### 3.3. Initial Animal Study with Onboard PET/CT Imaging

**Figure 6:**
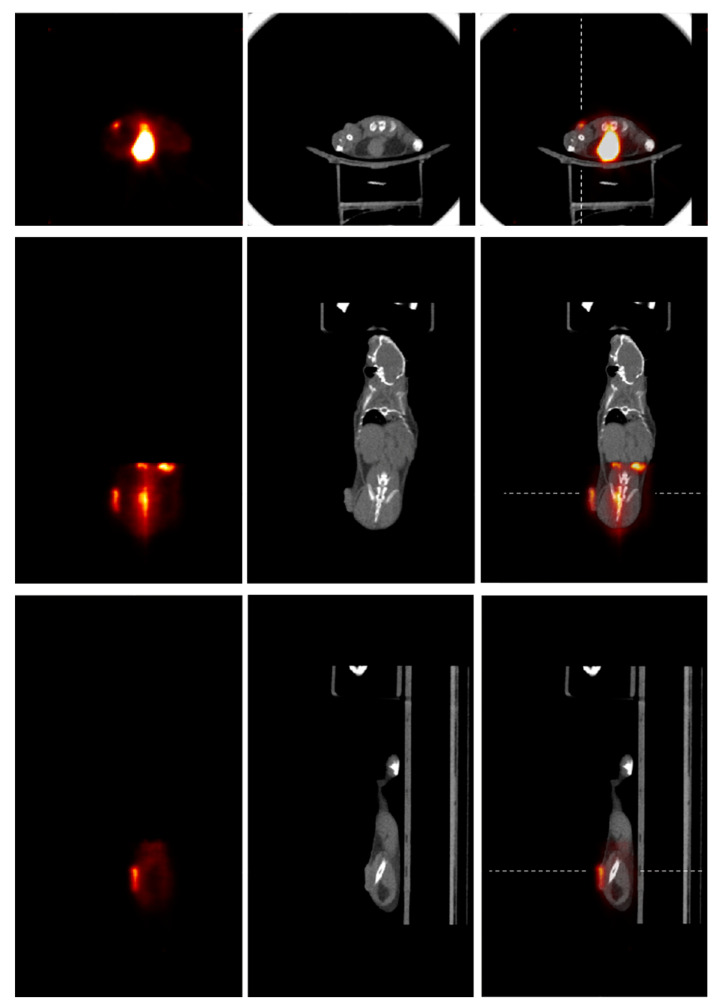
Images of the animal with trans-axial, coronal, and sagittal views are shown from the top to bottom panels, and [^18^F]FDG PET, CT, and PET/CT images are shown, respectively, from left to right within each panel. Dashed lines in PET/CT images show the positions where the slice profiles were drawn to display image intensities as those shown in Figure 7.

Figure 6 shows the onboard PET/CT acquired [^18^F]FDG PET, CT, and registered PET/CT animal images. The PET image was acquired with a one-bed position that covered the tumor volume within the PET FOV. The tumor can be clearly identified from the PET in all image slices. However, it is difficult to identify the tumor from the CT image. The registered PET/CT images provide the expected functional and anatomical information for tumor identification and boundary determination.

**Figure 7:**
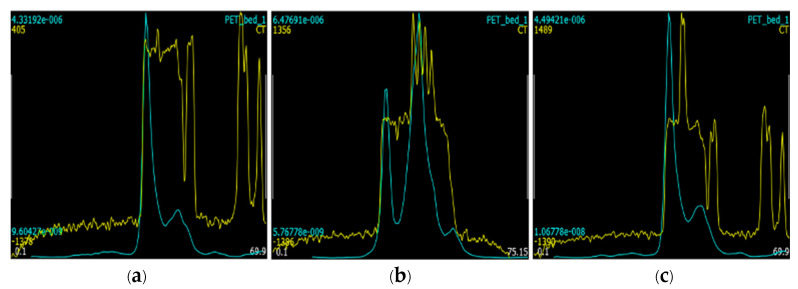
Intensity profiles of (**a**) trans-axial, (**b**) coronal, and (**c**) sagittal PET (blue) and CT (yellow) images at the selected slice positions as shown in Figure 6. PET and CT intensities are normalized for display. The additional numbers in the figures are the maximum and minimum intensity profile values of PET (blue) and CT (yellow) and the axis range (white).

Figure 7 shows the profiles across the PET/CT images at the positions as indicated in Figure 6. It is obvious that the outer edge of the tumor can be well determined from both the PET and CT images because the tumor was on the skin surface. However, the inner boundary of the tumor is rather difficult to be determined from the CT image, while it can be clearly determined from the PET image. It shows that PET/CT imaging can significantly improve tumor boundary determination, as anticipated, which should lead to enhanced RT accuracy with improved precision of beam targeting.

**Figure 8:**
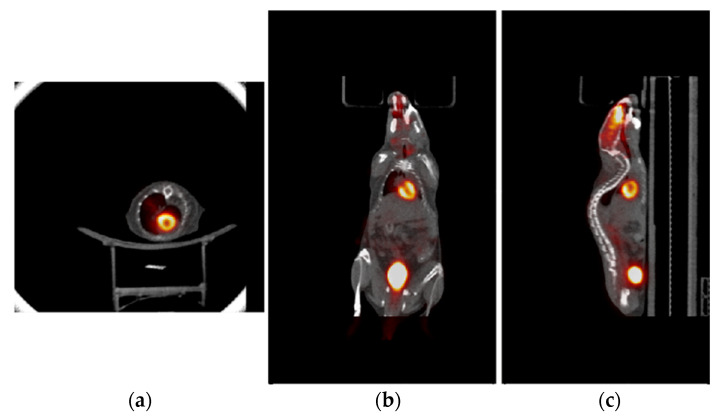
Whole-body animal PET/CT image with (**a**) trans-axial, (**b**) coronal, and (**c**) sagittal views. [^18^F]FDG PET data with three bed positions were sequentially acquired and their images were combined and registered with CT image.

Figure 8 shows the PET/CT whole-body images of the same animal with [^18^F]FDG PET acquisitions over three sequential bed positions. These selected slices show cardiac images with ventricle of the animal heart. It demonstrates the capability of acquiring onboard whole-body PET/CT animal images for image-guided preclinical RT study.

## 4. Discussion

Compared with a different approach with a pair of rotated PET detectors affixed to the CT gantry to provide PET/CT imaging [18], the integrated PET/CT/RT described in this study provides a stationary full-ring PET that can have substantial advantages which include significantly higher sensitivity and image quality, substantially shortened acquisition time with high counting rate, simplified operation without extra rotation of entire CT gantry solely for PET acquisition, more compatibility to the clinical PET/CT image-guided RT, and therefore practicality for routine preclinical RT research and translation to clinical applications [19,20,21,22].

In our study, there was no measurable PET detector performance degradation from the imaging acquisition with PET radioactive sources. However, PET detector performance is sensitive to the exposure of external X-ray radiations, and any such exposure could potentially degrade the detector performance by impacting its gain and background noise. That is because the interaction probability to the SiPM arrays, which are semiconductor photon sensors from low-energy X-ray photons, are much higher than that from the 511 keV coincidence gamma photons. The severity of the detector performance degradation is X-ray energy, intensity, and exposure time dependent. With a CT imaging associated collimator attached to the X-ray tube that shielded scattered X-ray photons, there was no measurable PET detector performance degradation after multiple CT acquisitions. However, without the shielding from a collimator, such as during the flood field X-ray radiation for CT detector calibration, there was measurable PET detector performance degradation. Therefore, the current prototype PET works fine with a routine CT imaging acquisition that requires a collimator anyway, but it is not suited to stay inside the CT/RT during X-ray radiations without a collimator attached, or a more rigorous X-ray shielding would be required. Fortunately, such flood field X-ray radiation is rarely performed.

In the current prototype development, two carbon-fiber flat plates with 53 cm long, 2.5 cm wide, and 2 mm thick, each plate was used as the rail to support the additional bed and guide its translational motion. One technical issue to be addressed in the next step is the attenuation of the rails to X-rays that led to visible CT image artifacts, such as those seen in Figure 5. Although these artifacts did not seriously impact the focused studies in this research on demonstrating the feasibility of integrated PET/CT/RT for onboard PET/CT imaging and they can be corrected with known attenuations, for practical application with streamed PET and CT acquisition processes and required image qualities, it is important to overcome this issue without an extra and lengthy data correction. The potential solutions include using lower density yet still mechanically strong material to construct the rails to minimize the attenuation to X-rays, or to extend the horizontal translational range of the existing 3D bed motion to avoid adding an additional translational bed. The latter is the best approach and is engineeringly feasible, although it will require the modification of the existing bed motion mechanical system.

The current prototype PET has a 3.5 cm axial FOV with one detector ring. Since detector and front-end readout electronics have modular designs, there are no fundamental technical challenges to extend the axial FOV by adding more detector rings to increase the system sensitivity. However, for the radiotherapy guidance with known tumor location and size, the current 3.5 cm axial FOV should be sufficient for most preclinical RT studies. The one detector ring also has the advantage of being light weight for PET integration, and it is also practical to acquire whole-body animal images with multiple bed acquisitions. On the other hand, the extended axial FOV with high sensitivity, improved image quality, and shortened scan time will be more suited for the imaging applications of diagnosis, RT monitoring, and therapy effectiveness assessment. Thus, a trade-off should be carefully considered for the pros and cons to extend the axial PET FOV.

Another potentially required improvement to the prototype onboard PET/CT integration is to minimize the number of signal transmission cables. Currently, there are 12 LVDS cables that are cumbersome to handle and install inside the CT/RT, and the number of cables will be increased with increased detector rings. Some inter-detector signal processing, multiplexing, and transmission needs to be investigated, particularly if a larger number of detectors will be implemented.

The PET performance with ambient temperature variation inside the CT/RT is another potential concern to be addressed. During our imaging and radiation study, the ambient temperature inside the CT/RT irradiator was increased ~1–2 °C after ~30 min since PET powered on, mainly due to the heat generated from PET FPGA electronics within the enclosed environment, but which stabilized after reaching the heat equilibrium. The PET gain and noise were also changed and stabilized accordingly. With appropriate energy window selection, PET imaging capability in our study was not affected. However, for routine applications with extended imaging time and repeated opening and closing of the irradiator enclosure, it is worth it to implement a mechanism to stabilize the ambient temperature inside the irradiator or adaptively adjust the detector voltage bias to stabilize the detector performance.

Although PET and CT images can be accurately registered as demonstrated, the dual-modality coordinates alignment is a lengthy and complex process. Different from a clinical PET/CT where both scanners are tightly integrated together, the prototype preclinical PET was inserted inside the CT/RT and fixed with metal bars; quite often the PET needed to be removed and be inserted again, which could lead to coordinates misalignment and performing a new alignment procedure. Thus, either an accurate and reliable PET insert method beyond the current approach or a fast and accurate coordinates alignment method is needed for robust and routine preclinical PET/CT image-guided RT applications.

With its compact size, light weight, and relatively large size animal port, the PET can also be integrated with other popular and latest animal CT/RT irradiators [23,24]. Besides hardware integration by affixing PET inside a CT/RT, it is also feasible and potentially advantageous to mechanically insert the PET inside CT/RT for PET imaging and to optionally move it outside CT/RT after the imaging. This approach can increase the flexibility in terms of using the insert PET and CT/RT separately and reduce the interference between them, potentially have PET and CT acquisitions with overlapped image FOV without moving the animal, and minimize or even eliminate the radiation shielding to PET. In addition, it will also minimize the PET temperature-dependent performance variation, as the entire PET imaging session will be conducted with open irradiator enclosure under a stable room temperature. On the other hand, the challenges with respect to the accurate alignment of the inserted PET to CT/RT stably and repeatably for routine applications will have to be addressed.

The future studies in this research will mainly include implementing and evaluating full data corrections and quantitative PET image processes, potentially integrating PET/CT acquisitions and processes under the same console, and most importantly guiding the treatment plan with the PET/CT functional and anatomical images and evaluating the effectiveness of this new paradigm of image guidance for preclinical radiation oncology research, such as the dosimetry evaluation including some guidelines for the target volume delineation and the calculation of CTV-PTV uncertainty interval depending on the possible sources of errors and uncertainties.

After all the above improvements, it is our belief that the animal PET/CT/RT can play a critical role in accelerating preclinical RT research and expanding the investigation of tumor radiation biology and the related clinical translation at the level driven by applying tumor functional images and information, which will permit researchers to directly understand the impact of radiation with different doses, target volumes, and fractionations to the tumor and normal tissues, and therefore develop the optimized treatment plan. By providing functional PET images to understand the intricate systematic biological effects of different radiation deliveries and exploring various novel RT approaches, the onboard PET/CT/RT may also play an important role in the forefront RT research and applications, such radiomics, immuno-radiotherapy, and ultrahigh dose rate (FLASH) radiotherapy [25]. For example, with the growing interest in preclinical PET radiomics studies [26], animal PET/CT/RT can provide a missing dataset of quantitative, biological image-guided radiotherapy to facilitate the radiomics analysis and modeling in preclinical studies. Additionally, it will also enable co-clinical radiomics investigations and potentially radiomics guided radiotherapy applications by comparing the clinical radiomics analysis and the preclinical outcome resulted from high-precision and biologically targeted animal radiation studies with a known disease model [27].

## 5. Conclusions

The first small animal integrated PET/CT/RT with the integration of a compact and lightweight stationary PET within a CT/RT has been developed and evaluated for its onboard PET/CT imaging capability. The initial study has shown that the prototype can achieve accurately registered onboard PET/CT phantom and animal images with normal PET and CT imaging conditions and acquisitions. The study demonstrated that an integrated PET/CT/RT can provide practical onboard functional/biological/molecular and anatomical image-guided preclinical radiation research with significantly improved accuracy of tumor delineation and radiation targeting, which will enhance the existing study, enable potentially new and breakthrough investigations, and ultimately expand and accelerate the radiation oncology research.

## Data Availability

No other data are available. All supporting data are presented in the manuscript.

## References

[1] Cheng X., Hu K., Yang D., Shao Y. (2021). A compact and lightweight small animal PET with uniform high-resolution for onboard PET/CT image-guided preclinical radiation oncology research. Phys. Med. Biol..

[2] Cheng X., Hu K., Shao Y. (2019). Dual-Polarity SiPM Readout Electronics Based on 1-bit Sigma-Delta Modulation Circuit for PET Detector Applications. IEEE Trans. Nucl. Sci..

[3] Cheng X., Hu K., Yang D., Shao Y. (2021). Field-programable-gate-array-based distributed coincidence processor for high count-rate online positron emission tomography coincidence data acquisition. Phys. Med. Biol..

[4] Shekhar R., Walimbe V., Raja S., Zagrodsky V., Kanvinde M., Wu G., Bybel B. (2005). Automated 3-dimensional elastic registration of whole-body PET and CT from separate or combined scanners. J. Nucl. Med..

[5] Marinelli M., Positano V., Tucci F., Neglia D., Landini L. (2012). Automatic PET-CT Image Registration Method Based on Mutual Information and Genetic Algorithms. Sci. World J..

[6] De Lombaerde S., Neyt S., Kersemans K., Verhoeven J., Devisscher L., Van Vlierberghe H., Vanhove C., De Vos F. (2017). Synthesis, in vitro and in vivo evaluation of 3β-[18F]fluorocholic acid for the detection of drug-induced cholestasis in mice. PLoS ONE.

[7] Lavely W.C., Scarfone C., Cevikalp H., Li R., Byrne D.W., Cmelak A.J., Dawant B., Price R.R., Hallahan D.E., Fitzpatrick J.M. (2004). Phantom validation of coregistration of PET and CT for image-guided radiotherapy. Med Phys..

[8] Vogel W.V., van Dalen J.A., Wiering B., Huisman H., Corstens F.H., Ruers T.J., Oyen W.J. (2007). Evaluation of Image Registration in PET/CT of the Liver and Recommendations for Optimized Imaging. J. Nucl. Med..

[9] Ghita M., Brown K.H., Kelada O.J., Graves E.E., Butterworth K.T. (2019). Integrating Small Animal Irradiators with Functional Imaging for Advanced Preclinical Radiotherapy Research. Cancers.

[10] Oderinde O.M., Shirvani S.M., Olcott P.D., Kuduvalli G., Mazin S., Larkin D. (2021). The technical design and concept of a PET/CT linac for biology-guided radiotherapy. Clin. Transl. Radiat. Oncol..

[11] Cheng X., Hu K., Xiong Z., Yang D., Shao Y. (2019). Initial performance evaluation of a compact add-on PET scanner for small animal PET/CT/RT: A rotating dual detector panel study. Proceedings of the 2019 IEEE Nuclear Science Symposium and Medical Imaging Conference (NSS/MIC).

[12] Rogasch J.M., Hofheinz F., Lougovski A., Furth C., Ruf J., Großer O.S., Mohnike K., Hass P., Walke M., Amthauer H. (2014). The influence of different signal-to-background ratios on spatial resolution and F18-FDG-PET quantification using point spread function and time-of-flight reconstruction. EJNMMI Phys..

[13] Gong S., O’Keefe G., Scott A. (2005). Comparison and Evaluation of PET/CT Image Registration. Conf. Proc. IEEE Eng. Med. Biol. Soc..

[14] Merlin T., Stute S., Benoit D., Bert J., Carlier T., Comtat C., Filipovic M., Lamare F., Visvikis D. (2018). CASToR: A generic data organization and processing code framework for multi-modal and multi-dimensional tomographic reconstruction. Phys. Med. Biol..

[15] Feldkamp L.A., Davis L.C., Kress J.W. (1984). Practical cone-beam algorithm. J. Opt. Soc. Am. A..

[16] Meikle S.R., Badawi R.D., Bailey D.L. (2005). Quantitative Techniques in PET, in Positron Emission Tomography: Basic Sciences.

[17] Cheng X., Hu K., Yang D., Shao Y. (2020). Design and development of a compact high-resolution detector for PET insert in small animal irradiator. Proceedings of the 2020 IEEE Nuclear Science Symposium and Medical Imaging Conference (NSS/MIC).

[18] Mikhaylova E., Brooks J., Zuro D.M., Nouizi F., Kujawski M., Madabushi S.S., Qi J., Zhang M., Chea J., Poku E.K. (2019). Prototype Small-Animal PET-CT Imaging System for Image-Guided Radiation Therapy. IEEE Access.

[19] Townsend D.W., Wensveen M., Byars L.G., Geissbuhler A., Tochon-Danguy H.J., Christin A., Defrise M., Bailey D.L., Grootoonk S., Donath A. (1993). A rotating PET scanner using BGO block detectors: Design, performance and applications. J. Nucl. Med..

[20] Bailey D.L., Young H., Bloomfield P.M., Meikle S.R., Glass D., Myers M.J., Spinks T.J., Watson C.C., Luk P., Peters A.M. (1997). ECAT ART-a continuously rotating PET camera: Performance characteristics, initial clinical studies, and installation considerations in a nuclear medicine department. Eur. J. Nucl. Med..

[21] Tarantola G., Zito F., Gerundini P. (2003). PET instrumentation and reconstruction algorithms in whole-body applications. J. Nucl. Med..

[22] Jones T., Townsend D. (2017). History and future technical innovation in positron emission tomography. J. Med Imaging.

[23] SmART+, *Small Animal Radiation Therapy (SmART) Systems*. Precision X-ray Irradiation, USA. https://precisionxray.com/systems/.

[24] SARRP, Small Animal Radiation Research Platform Xstrahl, USA. https://xstrahl.com/sarrp/.

[25] Van Dyk J. (2020). The Morden Technology of Radiation Oncology: A Compendium for Medical Physicists and Radiation Oncologists. Volume 4. Med. Phys. Intern J..

[26] Benfante V., Stefano A., Comelli A., Giaccone P., Cammarata F.P., Richiusa S., Scopelliti F., Pometti M., Ficarra M., Cosentino S. (2022). A New Preclinical Decision Support System Based on PET Radiomics: A Preliminary Study on the Evaluation of an Innovative ^64^Cu-Labeled Chelator in Mouse Models. J. Imaging.

[27] Roy S., Whitehead T.D., Li S., Ademuyiwa F.O., Wahl R.L., Dehdashti F., Shoghi K.I. (2022). Co-clinical FDG-PET radiomic signature in predicting response to neoadjuvant chemotherapy in triple-negative breast cancer. Eur. J. Nucl. Med. Mol Imaging.

